# The Rise of Post-truth Populism in Pluralist Liberal Democracies: Challenges for Health Policy

**DOI:** 10.15171/ijhpm.2017.19

**Published:** 2017-02-12

**Authors:** Ewen Speed, Russell Mannion

**Affiliations:** ^1^School of Health and Human Sciences, University of Essex, Colchester, UK.; ^2^Health Services Management Center, University of Birmingham, Birmingham, UK.

**Keywords:** Populism, Liberal Democracy, Post-truth Politics, Health Policy

## Abstract

Recent years have witnessed the rise of populism and populist leaders, movements and policies in many pluralist liberal democracies, with Brexit and the election of Trump the two most recent high profile examples of this backlash against established political elites and the institutions that support them. This new populism is underpinned by a post-truth politics which is using social media as a mouthpiece for ‘fake news’ and ‘alternative facts’ with the intention of inciting fear and hatred of ‘the other’ and thereby helping to justify discriminatory health policies for marginalised groups. In this article, we explore what is meant by populism and highlight some of the challenges for health and health policy posed by the new wave of post-truth populism.

## The Rise and Rise of Populism

Over recent years, some of the world’s most stable parliamentary democracies have witnessed the rise to prominence of so-called populist political movements and leaders. The election of President Trump in the United States and the United Kingdom’s vote to withdraw from the European Union (Brexit) have both been interpreted as a populist backlash against ‘out of touch’ political ‘elites’ and a challenge to the traditional institutions of liberal democracy. In this article, we unpack what is meant by populism and outline some of the implications and challenges of the upsurge in populism, in an era of ‘post-truth’ politics, for the health of populations and the implementation of health policy.

## It’s (not Only) the Economy, Stupid

Inglehart and Norris^1^ argue there are two broad demand side explanations for the rise in support for populist political parties, and recourse to populist policies among mainstream establishment parties (populism-lite) in liberal democracies. The most widely held view is that it is the result of increased *economic inequality* and growing social exclusion associated with post-industrial societies. From this perspective, neo-liberal austerity policies, the collapse of traditional manufacturing industry, technological change and global flows of labour, especially migrants and refugees, are claimed to have contributed towards increasing economic insecurity for large swathes of the population^2^ and this has helped to fuel popular resentment against traditional political institutions, particularly among the so-called left-behinds and the precariat.^3^ An alternative reading is the *cultural backlash* thesis, which views populism as not only an economic phenomenon, but also a counter revolutionary retro-backlash against successive waves of progressive cultural change since the 1970s, which have helped to foster greater social tolerance of diverse lifestyles, religions and cultures. The argument made is that large segments of the population, particularly older people, white men, and those with less formal qualifications, resent the displacement of their traditional social values and this creates a dissatisfied pool of potential voters who are susceptible to seductive populist appeals that offer a return to a ‘golden age’ of national identity and traditional social values. Recent empirical work across 31 European countries found support for the cultural backlash thesis and concluded that “the orthogonal pull of cultural politics generates tensions and divisions within mainstream parties, as well as allowing new opportunities for populist leaders on the left and right to mobilise electoral support.”^1^ But do these two perspectives adequately explain populism? Or is it more complicated than that?

## What Is Populism: Power to the People?

Populism is one of the most contested concepts in the social sciences, is riddled with paradox and fraught with rival interpretations. Politically, it is neither ostensibly of the right, middle or the left. The vagueness of populism as a concept, and as a political strategy, is what makes it at once both analytically slippery and politically useful.^4^ According to Weyland^5^ populism has been defined as a “political strategy through which a personalistic leader seeks or exercises power based on direct, unmediated, uninstitutionalized support from large numbers of mostly unorganized followers” (p.14). Albertazzi* and McDonnell,*^6^ interpret it as an ideology that “pits a virtuous and homogeneous people against a set of elites and dangerous ‘others’ who are together depicted as depriving (or attempting to deprive) the sovereign people of their rights, values, prosperity, identity, and voice” (p. 3). For Mudde,^7^ populism represents only a ‘thin ideology,’ that merely sets up a hypothetical confrontation between the will of the ‘pure’ people versus a corrupt elite, with populists claiming that they alone represent the people and their true interests. In reality, the political initiative emanates from the personality – and whims – of the leader, and not the popular voting base. The implication is that this thin ideology can be appended to a range of “thick” ideologies with more mature political logics such as socialism or nationalism in order to pursue wider political agendas. This vagueness as a precondition to constructing relevant political meanings is demonstrated by the seeming lack of contradiction between left-wing populists championing the ‘people’ against an economically privileged neo-liberal business elite and right-wing populists championing the ‘people’ against an elite accused of favouring a third group of their choice (usually based on unapologetic religious bigotry, racism and misogyny). The more ethnocentric the conception of the ‘people’ the more xenophobic the positioning against the ‘other.’ Therefore, the key distinction between right-wing and left-wing populism is not whether they ostracise, but whom they ostracise. As populism concerns only the antagonistic relationship between the people and the elite, who is considered to be the elite, or the people, depends on the political orientation of the populist. Nonetheless, it is important (and no doubt reassuring for many) to note that the power base of populist leaders is necessarily fragile. As exemplified by Trump, populist leaders often emerge as ‘outsiders’ and are not well-aligned with established interest groups or embedded in the tight-knit networks of normal party politics, and therefore, cannot depend on their continuing support in times of trouble. As the theatrical machismo of vainglorious populist leaders fades and their policies ultimately fail, so does their appeal. These core attributes of populist politics and the personality traits that typify populist leaders have profound implications for the health of populations and the design and implementation of national (and international) health policies, and it is to these that we now turn.

## The Challenge of Evidence Informed Health Policy in a Post-truth World

Healthcare has benefitted enormously from international cooperation and agreements that allow the free flow of people, capital, goods, and information.^8,9^ Populism, on the other hand, is concerned with national protectionism which limits international cooperation and movement.^6^ A populism built on ‘walls’ and fear of ‘the other’ (for Trump read Mexicans and Muslims, for Brexit read immigrants from Eastern Europe and Syrian refugees), discriminates against certain sub-sections of the population and exacerbates existing national (and global) health inequalities. Populist leaders pursing such policies typically try to avoid established institutional checks and balances (including the professionalised civil service) and seek to implement public policies at more pace and scale than the traditional bureau-incrementalistic approaches associated with liberal-democratic governments. This is clearly-evident in President Trump’s use of Twitter as a media platform to announce new policies and in so doing by-passing the professional experts and civil service. But swift reforms may come at the expense of good policy design and mass support, especially as populist policies tend to be shaped more by the personal whims and prejudices of a demagogue than underpinned by a secure evidence base. In the United States, a clear example of this relates to changes to policy in the realm of reproductive rights, with legislation already proposed which seeks to limit access to abortion services. Similarly, the repeal of the Affordable Care Act may have very real consequences in terms of restricting access to contraception and other birth control services.There are also concerns around the so-called conscience laws which enable individuals and companies to use conscience objections against charges of sexual orientation discrimination.^10^ In the United Kingdom, there have been calls to introduce charging mechanisms for ‘health tourists,’ with the effect that overseas patients are required to pay upfront for their care. However, amid all the political clamour and rhetoric around ‘freeloading’ health tourists, it should not be lost that a greater principle is at stake – the introduction of a formal charging mechanism into a Beveridge based health system.^11^ Indeed, some hospitals in the United Kingdom have already started to charge for elective surgery,^12^ even though patients have already paid for access to care via direct taxation. Here we see the potency of populist appeals, where the invocation of the freeloading ‘other’ can be used to justify a fundamental change to the principle of universal healthcare and ultimately the dismantling of the National Health Service (NHS).^13^

Terms such as ‘post-truth’ and ‘fake news’ have been used to describe the rapid transformations to the substance of populist political discourse, both in relation to the election of president Trump and the Brexit campaign. As defined by the Oxford English dictionary (which made it the 2016 international word of the year) post-truth “relates to or denotes circumstances in which objective facts are less influential in shaping public opinion than appeals to emotion and personal belief.” And this clearly links to sociological approaches since the 1980s which have explored the ways in which developments in mass communication technology have created a sense of what Baudrillard^14^ described as ‘hyperreality’ – the inability to distinguish the real from the false and a postmodern condition, where even supposedly hard economic evidence can be contested.^15^ Populist politicians’ reliance on assertions that appear true, but have no basis in fact, creates a false view of the world, not with the intention of convincing the elites that they are right, but in reinforcing prejudices among their targeted pool of potential supporters. In the modern age of social media, fear, rumour and gossip can spread alarmingly fast with feelings and emotions often carrying more weight than facts and evidence. Charismatic leaders spread a populist mood which creates an additional emotional hook and which distinguishes populist political rhetoric from conventional politics. A common rhetorical device used in populist post-truth politics is the repetition of a dominant motif – which may not be based on any reliable evidence. For example, during the British EU referendum campaign, *Vote Leave* made repeated use of the claim that EU membership cost £350 million a week, but this claim was rejected in fact-checks undertaken by BBC News and several independent experts. Indeed, this provoked the notorious rebuke by the pro-leave Secretary of State for Justice, that “people in this country have had enough of experts.”^16^ It is not difficult to see that the disdain for policy experts by politicians pursuing populist policies, may result in poorly designed and implemented health policies with potentially serious dysfunctional consequences. Noveck aligns the recent surge in support for populism with the rise of a professional ‘expertocratic’ political class which has served to disenfranchise ordinary citizens from democratic decision making.^17,18^ But as she points out, expertise is clearly widely distributed in society, with citizens expert in everything from restaurant reviews to medical advising. The challenge in post-truth societies is to harness the potential of new technology to support more participatory styles of involvement in public affairs. These include taking advantage of the opportunities offered by new technological services such as crowdsourcing, ‘open source’ systems and Bazaar forms of citizen governance.^19^ Such approaches help to challenge the notion, promulgated in populist discourse, that expertise and wisdom are limited to professional bureaucrats and elite institutions.

## What’s Wrong With Being Popular?

The recent upsurge in support of populism is challenging the historical divide between the political left and right and a new cleavage is opening up between those clinging to conventional approaches to politics and those who are challenging establishment institutions with the lure of populist appeals. There are clear parallels with the events in Europe in the 1930s, with populist claims of putting the people first, while promoting division and turning people against one another. But there are also some key differences. Although populist leaders still use mass rallies and bombastic speeches, this new wave of discriminatory populism is underpinned by a post-truth politics which is using social media (the Trump Tweet) as a mouthpiece to peddle ‘fake news’ and circulate ‘alternative facts’ with the specific intention of shaping voter opinion and exciting emotions through inciting fear and hatred of the ‘other.’ There are no simple solutions to these concerns. But specifically, in relation to healthcare, strategies need to include challenging all forms of discrimination that limit access to services for marginalized social groups, as well as harnessing the power of new media technology to foster better citizen participation and involvement in important decisions that affect their health and healthcare. There can be little doubt that the ascendancy of a discriminatory populist politics will serve to widen existing inequalities in society, identifying categories of the deserving and undeserving ill. In such times, it is a pressing necessity that health policies in liberal democracies continue to offer a breadth of coverage that ensures parity of access, based on the rigorous application of research evidence, underpinned by robust processes of democratic engagement.

## Ethical issues

Not applicable.

## Competing interests

Authors declare that they have no competing interests.

## Authors’ contributions

Both authors contributed equally to the writing of this article.

## Authors’ affiliations

^1^School of Health and Human Sciences, University of Essex, Colchester, UK. ^2^Health Services Management Center, University of Birmingham, Birmingham, UK.
